# Supplementary material for: Capturing protein communities by structural proteomics in a thermophilic eukaryote
Source: Mol Syst Biol. 2017 Jul 25;13(7):936. doi: 10.15252/msb.20167412 (PMC5527848; doi:10.15252/msb.20167412)
Supplement: Supplementary file 1 — Appendix [file MSB-13-936-s001.pdf]

Appendix

Title: Capturing protein communities by structural proteomics in a thermophilic eukaryote

Authors: Panagiotis L. Kastritis[1†], Francis J. O'Reilly[1,2†], Thomas Bock[1†], Yuanyue Li[1], Matt Z. Rogon[1], Katarzyna Buczak[1], Natalie Romanov[1], , Matthew J. Betts[3], Khanh Huy Bui[1,4], Wim J. Hagen[1], Marco L. Hennrich[1], Marie-Therese Mackmull[1], Juri Rappsilber[2,5], Robert B. Russell[3], Peer Bork[1], Martin Beck[1*], Anne-Claude Gavin[1*]

(**Running title:** Structural systems biology of lysates)

Affiliations:

[1] European Molecular Biology Laboratory, Structural and Computational Biology Unit, 69117 Heidelberg, Germany

[2] Chair of Bioanalytics, Institute of Biotechnology, Technische Universität Berlin, 13355 Berlin, Germany

[3] Cell Networks, Bioquant, Im Neuenheimer Feld 267 & Biochemie Zentrum Heidelberg, Im Neuenheimer Feld 328, Heidelberg University, 69120 Heidelberg, Germany

[4] Department of Anatomy and Cell Biology, McGill University, Montreal, Quebec H3A 0C7, Canada

[5] Wellcome Trust Centre for Cell Biology, School of Biological Sciences, University of Edinburgh, Edinburgh EH9 3BF, United Kingdom

Corresponding authors

* Correspondence should be addressed to martin.beck@embl.de and gavin@embl.de

Additional Footnotes

† Equally contributing authors.

This PDF file includes:

Appendix Supplementary Methods (pages 2-13)
Appendix Figs. S1 to S15 (pages 14-33)

Appendix Supplementary Methods

Culture growth

Chaetomium thermophilum var. *thermophilum* La Touche 1950 cultures (strain type DSM1495, German Collection of Microorganisms and Cell Cultures) were grown in 400 ml standard LB medium (50°C; 10% CO_2 supplemented atmosphere) with an orbital shaking unit (60 rpm, shaking diameter: 50 mm) for 48 h.

Culture lysis

PBS-washed fungal cultures were freeze-ground in liquid nitrogen. Approximately 12 g of freeze-grinded culture was lysed into 20 ml lysis buffer (100 mM HEPES pH 7.4, 95 mM NaCl, 5 mM KCl, 1 mM $MgCl_2$, 1 mM EGTA, 1 mM DTT, 10 ug/ml DNAse, pefabloc 2.5 mM, E-64 40 uM, Bestatin 130 uM, Aprotinin 0.5 uM, Leupeptin 1 uM, pepstatin A 60 uM) in a Fastprep FP120 at 4ºC (3 x 6.5 mps shaking speed with 2 mins rest on ice). Lysate was cleared of debris by centrifugation at 100,000 *g* for 45 min. Native protein complexes were further concentrated by spin filtration using a 100,000 Da cut-off Amicon Ultra centrifugal filter unit (approx. 30 min).

Size exclusion chromatography

For protein co-elution analysis, 100 µl of concentrated lysate (approximately 30 mg/ml) was separated by a Biosep SEC-S4000 (7.8 x 600) size exclusion column on an EttanLC (HPLC) system running at 0.25 ml/min in 100 mM HEPES pH 7.4, 95 mM NaCl, 5 mM KCl, 1 mM $MgCl_2$. 250 µl fractions were collected. Fractions were subjected to mass spectrometry (MS), electron microscopy (EM), or cross-linking analysis.

Preparation of protein samples for mass spectrometry

Protein amount was determined by BCA assay for each fraction. For protein co-elution analysis, proteins in each fraction were denatured in 4 M urea buffer containing 0.2% (w/v) Rapigest® detergent. Carbamidomethylation of cysteines was performed prior to protein digestion by adding 10 mM DTT at 37°C for 30 min followed by 15 mM IAA at room temperature in the dark for 30 min. Proteins were digested first using 1:100 (w/w) LysC (Wako Pure Chemical Industries) at 37°C for 4 h and second with 1:50 (w/w) trypsin (Promega) at 37°C for 12 hours after diluting the urea concentration to 1.5 M. Acidified samples (10% trifluoroacetic acid, vol/vol) were desalted using Macro SpinColumns (Harvard Apparatus) according to the manufacturer's procedure. Purified peptide mixtures were dried in a vacuum concentrator and stored at -20°C until further use. 30 fractions from the first biological replicate were pooled and dimethyl labeled (heavy) to generate a spike-in standard. These were added to all fractions dimethyl labeled (light) in a 1:1 ratio of peptide abundance. Labeling was done in solution as described (Boersema et al, 2009).

Simplification of cell lysate by anion exchange

Lysate was produced as above and passed through a HiTrap DEAE FF column (GE Healthcare) 0.25 ml/min in 100 mM HEPES pH 7.4, 95 mM NaCl, 5 mM KCl, 1 mM $MgCl_2$. Flow-through was collected and concentrated by spin filtration using 100,000 Da cut-off Amicon Ultra centrifugal filter unit. Sample was then separated by size exclusion chromatography and prepared for mass spectrometry analysis as above.

Mass spectrometry analysis

For protein co-elution, size exclusion chromatography fractions were analyzed in biological triplicates. Peptides mixtures were separated with a BEH300 C18 (75 μm x 250 mm, 1.7 μm) nanoAcquity UPLC column (Waters) using a stepwise 75 min gradient from 3% to 85% (v/v) acetonitrile in 0.1% (v/v) formic acid at a flow rate of 300 nl/min. The connected LTQ-Orbitrap Velos Pro instrument (Thermo Scientific) that was operated in data-dependent mode performing one survey MS1 scan followed by up to 20 (TOP20) collision-induced dissociation (CID)-based fragmentation MS/MS scans of the highest abundant ions available. Only charge states >1 were allowed for fragmentation. Important MS settings were: m/z range: 375-1600; resolution: 30,000 FWHM; AGC: 10^6; maximum ion time: 500 ms. Important MS2 settings were: minimum signal threshold: 150; dynamic exclusion time: 30 s; isolation width: 2 Da; normalized collision energy: 40; activation Q: 0.25; AGC: 30,000; maximum ion time: 50 ms, The mass spectrometry proteomics will be deposited to the ProteomeXchange Consortium (Vizcaino et al, 2014) via the PRIDE partner repository.

Finally, simple calculation of the percentage of proteins identified in U2OS and HeLa cells was performed. For HeLa cells, Nagaraj et al (Nagaraj et al, 2011) report 10,255 proteins; Kristensen et al. (Kristensen et al, 2012) found 1,961 proteins in their SEC experiment (19.1 % of total proteome), using the same column (see above). Similarly, for U2OS cells, Beck et al (Beck et al, 2011) identified 10,006 proteins; Kirkwood et al (Kirkwood et al, 2013) report 2,926 proteins identified in the larger size fractions (29.2% of total proteome).

Mass spectrometry data search, protein identification and analysis

MS raw data for each fraction were searched against the *Chaetomium thermophilum* protein database including common protein contaminants using the MaxQuant search engine (version 1.305) (Cox & Mann, 2008). Important search parameters were: missed cleavages, 2; variable modifications, oxidation (M), N-acetylation (protein N-terminus); multiplicity, 2, allowing for light and heavy dimethyl labeling; static, carbamidomethylation (C); matching between runs, yes. Proteins and peptides were filtered for 1% FDR using target-decoy-based reverse database search. Further filters applied were: protein score, equal or higher than 60; PEP, <0.05; number of unique peptide counts, equal or higher than 2. Major protein ID was used for identified protein groups. Label-free quantitation of protein intensities was deemed more efficient than labeled spike-in standard quantitation. Thus, protein intensities for the biological triplicates (dimethyl light channel) were exported as iBAQ scores as determined by the MaxQuant software.

Preparation of cross-linking samples for Xi and xQuest searches

For cross-linking of *C. thermophilum* protein complexes, consecutive fractions were pooled into three-fraction pools. For the xQuest pipeline, native protein complexes in fraction pools were cross-linked using 0.5 mM isotope-labeled disuccinimidyl suberate (DSS, Creative Molecules) at 35°C, 600 rpm, for 30 min. The cross-linking reaction was quenched by the addition of 100 mM ammonium bicarbonate at 35°C, 600 rpm, for 10 min. For the Xi pipeline native protein complexes in fraction pools were cross-linked using 1:1 protein mass: cross-linker (BS3 from Thermo Scientific) mass ratio at 4°C, 600 rpm, for 2 hours min. The cross-linking reaction was quenched by the addition of 100 mM ammonium bicarbonate at 4°C, 600 rpm, for 30 min.

An additional cross-linking experiment was performed using a preperative SEC column for the xQuest pipeline: 2 ml of an approximately 12.5 mg/ml solution was injected onto a HiPrep 26/60 Sephacryl S-400 HR column at 0.2 ml/min and 300 µl fractions were collected. Consecutive fractions were pooled into seven-fraction pools and cross-linked using DSS. These were individually concentrated to 100 µl in order to achieve a concentration of approx. 0.5-1 mg/ml.

Cross-linked protein complexes were denatured by addition of 4 M Urea/0.1% 0.2% (w/v) Rapigest® and subjected to carbamidomethylation and digestion as described above. Purified peptide mixtures of cross-linked samples were dried in a vacuum concentrator and stored at -20°C until further use. Cross-linked peptides derived from each fraction pool were enriched using peptide gel filtration as described previously (Leitner et al, 2014). In brief, samples were reconstituted (30% (v/v) acetonitrile / 0.1% (v/v) trifluoroacetic acid) and fractionated using a Superdex Peptide PC 3.2/30 column (GE) on an Äktamicro LC system (GE) at a flow rate of 50 µl/min. Fractions eluting between 0.9 and 1.4 ml based on the 215 nm UV absorbance profile were collected (3-4 fractions total for each of the original fraction pools) and dried in a vacuum concentrator. Fractions were reconstituted in 20-40 µl buffer containing 5% (v/v) acetonitrile and 0.1% (v/v) formic acid prior to mass spectrometry analysis

Mass spectrometry and data analysis with xQuest of cross-linked samples

For cross-linking analysis, a total of 24 peptide gel filtration fractions were analyzed in technical duplicates with the same settings as described above, except a stepwise 60 min gradient was used and the minimum signal threshold was set to 100.

Mass spectrometry and data analysis with Xi of cross-linked samples

Samples were analyzed using a UltiMate 3000 Nano LC system coupled to an Orbitrap Fusion Lumos Tribrid mass spectrometer equipped with an EasySpray Source (Thermo Fisher Scientific, San Jose, CA). Mobile phase A consisted of 0.1% formic acid in water, mobile phase B of 80% acetonitrile, 0.1% formic acid and 19.9% water. Peptides were loaded onto a 500 mm C-18 EasySpray column (75 µm ID, 2 µm particles, 100 Å pore size) with 2% B at 300 nl/min flow rate for 11 min and eluted at 300 nl/min flow rate with a linear gradient from 2% - 40% B over 139 min.

Raw files were preprocessed with MaxQuant (v1.5.4.1), using the partial processing until step 5. Resulting peak files (APL format) were subjected to Xi (Giese et al, 2016), using the following settings: MS accuracy, 6 ppm; MS/MS accuracy, 20 ppm; enzyme, trypsin; max. missed cleavages, 4; max. number of modifications, 3; fixed modification: carbamidomethylation on Cysteine; variable modifications: oxidation on

Methionine; cross-linker: BS3 (mass modification: 109.0396 Da). Variable modifications of the cross-linker (‘BS3-NH2’, mass modification: 155.0946 Da; ‘BS3-OH’, 156.0786 Da) and looplinks (‘BS3-loop’, 138.0681 Da) were allowed. BS3 was assumed to react with Lysine, Serine, Threonine, Tyrosine or the protein N-terminus. FDR was estimated using at residue pair level using XiFDR (Fischer & Rappsilber, 2017).

Prediction of co-eluting proteins

Proteins detected in more than one biological replicate and with a positive identification in more than three consecutive fractions were retained for co-elution analysis. Data in each fraction were averaged across biological replicates and each chromatogram was correlated point by point with each other chromatogram to generate a Pearson Correlation Coefficient. A cross-correlation score was then derived as a threshold for protein co-elution discovery, and calibrated by an assembled benchmark of protein complexes with known molecular structures. An algorithm is available from the authors upon request.

Ortholog mapping

Orthologous protein groups within *C. thermophilum*, *C. globosum*, *Neurospora crassa* and 20 related fungi (pezizomycotina) were generated using the in-house eggNOG pipeline and these pezizomycotina orthologuous groups were mapped to fungal orthologous groups (fuNOG) from eggNOG (Jensen et al, 2008). If fuNOG groups had at least two members of the pezizomycotina orthologous groups in them and any *S. cerevisiae* protein in the same group, it was assigned as an ortholog of *C. thermophilum*.

Protein interaction prediction using known 3D structures of homologs

We used the Mechismo (Betts et al, 2015) pipeline (minus filtering for experimentally determined interactions) to predict *C. thermophilum* protein-protein interactions based on known 3D interaction structures of homologs, and scored the resulting interactions with InterPreTS (Aloy & Russell, 2002).

Protein interaction prediction

For the computation of the network and subsequent clustering, the R programming language was used with related packages (Liaw & Wiener, 2002; Williams, 2011). Existing protein-protein interaction knowledge was mined from String (v.9.1) (Franceschini et al, 2013) by extracting all known interactions between Chaetomium orthologs in *S. cerevisiae*. String scores were re-processed to remove physical association evidence and retaining all other experimental evidence (e.g. genetic interactions). Cross-correlation co-elution (CCC) scores were filtered for scores above 0. The distributions of these three datasets and their overlap are found in **Appendix Fig. S7**. Weighted biochemical interaction data from the co-elution study (936,056 interactions containing 330,330 positively scored (>0) experimental interactions) were combined with interface predictions from Mechismo (744, Z-score) and existing knowledge derived from String (75,914) using a Random Forest (RF) classifier and a manually curated training set of reference interactions to filter out spurious connections and infer a network of high confidence, in a similar manner to (Havugimana et al, 2012). As a result, no interaction in the final data exists without a foundation of an experimentally derived score.

A benchmark set of known protein complexes was assembled from the homologous PDB complexes and AP-MS-derived core *S. cerevisiae* complexes (Benschop et al, 2010) (**Dataset EV2**). This benchmark contains 5,926 true positives (TP) and 99,837 true negatives (TN), assumed from inter-complex interactions. A machine learning training set was created from it by randomly subsampling from the positive and negative interactions to achieve a balanced, 1:1 ratio of TP:TN (~10,000 interactions), and split into three independent sets for learning, validation, and testing (70/15/15% respectively). The performance of the model was validated on the out-of-bag (OOB) sample, such that each of the 500 trees was constructed using a different bootstrap sample from the original data to get an unbiased estimate of the test set error. Approximately 63% of the available data were used for the tree construction, leaving the remaining 37% for testing [the OOB data]. Our RF Model has yielded an OOB error rate of ~8% and a consistent ROC of ~97% across independently evaluated sets (**Appendix Fig. S7**). Quality was evaluated using the OOB error rate, ROC, as well as the mean decrease accuracy and mean decrease Gini as measures of predictor variable importance in reducing classification error and partitioning the data into defined classes respectively. The resulting RF model was applied to the unclassified data (322,672 positive co-elution experimental scores). RF-produced probability score distribution was evaluated and a cut-off for significance of 1 Standard Deviation (SD) was selected for predicted class one interactions (resulting in an RF probability threshold of >= 0.85) (**Appendix Fig. S7**). The retained network contained 679 proteins connected with 6,002 unique, un-directed, non-duplicated edges (**Dataset EV3**). The quality of the RF-derived network was evaluated using a randomized Erdős–Rényi model (Erdős & Rényi, 1959) (for the same number of nodes and edges). Calculated network centralities show that the RF network is a non-random, scale-free, real-world network (**Appendix Fig. S7**). The degree distribution follows the power law with a correlation of 0.8 and R-squared of 0.7, compared with the randomized network correlation of -0,044, and R-squared = 0.003 and the global clustering coefficient of 0.5 vs. random networks' 0.026. Network was visualized using Cytoscape v3.3 (Shannon et al, 2003) (**Appendix Fig. S7**).

Community detection

Protein complexes were inferred using the ClusterONE algorithm (Clustering with Overlapping Neighborhood Expansion) (Nepusz et al, 2012). ClusterONE assumes that protein complexes appear as densely connected regions within the inferred interaction network, and proteins may belong to multiple complexes, thus the regions may overlap. Complexes are discovered by growing multiple clusters from seed proteins, independently of each other, and the greedy algorithm attempts to maximize the cohesiveness of the complex (Nepusz et al, 2012). The input network weighted by RF probability was clustered with the following parameters: node penalty value=2.0; cluster density threshold=0.3; haircut threshold=0.3; seeding from all nodes; multi-pass; weights=RF probability. All parameter combinations were varied and optimized to yield the highest Maximum Matching Ratio (a measure of how accurately the predicted complexes represent the reference set). MMR alongside of the positive predictive value (PPV), clustering-wise sensitivity (cws), and geometric accuracy (acc) (Brohee & van Helden, 2006) formed a quality benchmark evaluating how well the result recovers the benchmark set of complexes. Clustering yielded a total of 92 significant clusters (p-value

<0.1). Removal of redundancy (merging clusters containing ≥50% overlapping subunits) reduced this to 65 clusters. Clusters with a clustering coefficient (transitivity) of 0 were removed, as we expect protein complexes to show higher density (connectivity) among members (Wasserman & Faust, 1994). Three clusters containing proteins eluting in the final fraction were not considered due to their incomplete elution peaks. The final 48 high-confidence clusters are shown in **Dataset EV4**.

Structure prediction of proteins participating in high-molecular weight assemblies

Prediction of the structure of all 1,176 identified proteins was performed with iTASSER v4.2 (Yang et al, 2015). The top predicted model was selected according to its respective c-score,(Roy et al, 2010). Details for model quality for those with >30% of sequence identity and coverage are shown in **Appendix Fig. S2**; detailed coordinates for the models are available from the authors upon request.

Protein complex assignment using the Protein Data Bank

Each of the 1,176 proteins found in total in all three biological replicates were manually submitted to the NCBI BLAST server (http://blast.ncbi.nlm.nih.gov/Blast.cgi) and searched against the PDB (www.pdb.org). A threshold of 30% of sequence identity was assigned. Decision on the assembly was taken after back-BLASTing the rest of the subunits, if any, of the PDB structure to the *C. thermophilum* proteome. All results are included in **Dataset EV2**.

Calibration of cross-linking ld score using structural models

For proteins for which cross-links were identified, cross-linking distances on the structure were calculated with Xwalk (Kahraman et al, 2011). Furthermore, protein models with known complex states were modeled according to their template protein complex by superimposing the *C. thermophilum* atomic models on top of their structural homologs with PyMOL v1.2 (www.pymol.org). Data is included in Dataset EV2 regarding homology.

Modeling of protein interfaces using cross-linking data

The HADDOCK webserver (guru access) was used (de Vries et al, 2010; van Zundert et al, 2015). Missing side-chains were properly built and the interface of the complex was optimized using the OPLS force field (Jorgensen & Tirado-Rives, 1988) and non-bonded interactions were calculated using a cut-off of 8.5 Å. Electrostatic energy (E_{elec}) was calculated by a shift function, while a switching function (between 6.5 and 8.5 Å) was used for the van der Waals energy (E_{vdw}). Desolvation energy was calculated by implementing empirical atomic solvation parameters (Fernandez-Recio et al, 2004). All calculations were performed with HADDOCK version 2.1/CNS version 1.2 (Brunger, 2007; Dominguez et al, 2003). Cross-linking data were implemented as interaction restraints, set to have an effective (and maximum) Cα-Cα distance of 35.2 Å, whereas the minimum distance was only defined by energetics. This distance was selected due to the maximum Cα distance that the DSS cross-linker may have, when cross-linking Lys side-chains. For docking calculations, the standard HADDOCK protocol was used (Dominguez et al, 2003). Standard HADDOCK scoring for it0 was applied to select the 200 top-ranking structures that awere subsequently passed onto the next docking steps

(semi-flexible simulated annealing and final refinement in explicit water). Finally, clustering at 7.5 Å was performed. Parameter files for each run performed in this study are available from the authors upon request.

Negative-stain electron microscopy and 2D class averaging

Samples were directly deposited on glow-discharged (60 sec) Quantifoil®, type 300 mesh grids and negative-staining with uranyl acetate 2%(w/w) water was performed. In short, after sample was applied, it was rinsed twice with distilled water after 30 sec and uranyl acetate was then applied for 60 sec. After blotting, grids were left to air-dry and subsequent imaging at the FEI Morgagni 268(D) was performed for each fraction. Recording of data was performed with a side-mounted 1K CCD Camera (SIS). After data acquisition (pixel size=7.1 Å), E2BOXER was used for particle picking with a box size of 80X80 pixels. In total, 37,424 particles were picked out of 30 fractions, resulting in 1,170±827 particles per fraction. Class averaging was performed using RELION 1.2 (Scheres, 2012a; Scheres, 2012b). Briefly, 20 classes per fraction were set for 2D classification. Cross-correlation of final class averages was performed using an in-house MATLAB script. The algorithm is available from the authors upon request.

ctFAS enzyme preparation and vitrification

Three fractions derived from SEC enriched in ctFAS (according to quantitative MS data) were pooled and subsequently visualized for structural integrity. ctFAS was ~50% enriched (see **Appendix Fig. S14**) and overall protein concentration was determined to be ~40 ng/μL. Samples were then deposited on glow-discharged (60 sec) carbon-coated holey grids from Quantifoil®, type R2/1. A FEI Vitrobot® was used for plunge-freezing. In short, humidity was set to 70%, temperature to 293° K, blotting and drain time to 3 and 0.5 sec, respectively. Sample volume applied was 3 μL and blot offset was set to -3 mm.

Image acquisition

The vitrified samples were recorded on a FEI Titan Krios microscope at 300 kV and automatic image acquisition was performed with FEI EPU software. Pixel size was set to 2.16 Å and an FEI Falcon 2 camera was used in movie mode. The total number of frame groups was seven and dose applied per frame group (e–/Å^2/sec) was set to 4/4/4/4/4/4/24, respectively. Total dose applied was summed to 48 e–/Å^2, but the last frame was used only for particle picking. Magnification was set to 75000X, whereas defocus ranged between 0.6 and 3.0 μm. A total number of 13,419 micrographs were acquired in 21 hours (1 frame/ 6 sec; 1 movie/42 sec).

Data processing and 3D reconstruction

Motion correction was applied to acquired micrographs (Li et al, 2013) leading to 1,917 summed micrographs. E2BOXER was used for particle picking with a box size of 256X256 pixels. 7370 particles were selected from 1597 micrographs (4-5 particles/image). For CTF correction, CTFFIND from the Grigorieff lab was used (Rohou & Grigorieff, 2015). The RELION 1.2 package (Scheres, 2012a; Scheres, 2012b) was then used for 2D class averaging, 3D classification and 3D reconstruction of the density map. Briefly, 20 classes were set for 2D classification and, after visual inspection, eight

were selected for further processing that included 4,898 particles. 3D classification was set to five classes, performed without imposing symmetry, using the cerulenin-inhibited yeast FAS as an initial model (Gipson et al, 2010), low-pass filtered to 60 Å. 3,933 particles were successfully classified in a single class (**Appendix Fig. S14**) and this was subsequently used for reconstruction. 3D reconstruction was performed using the same initial model but applying D3 symmetry and underwent 25 interactions, showing convergence. Significant dependency of the resolution according to masking was observed (**Appendix Fig. S14**), but default Gaussian mask from RELION 1.2 leads to a calculated resolution (Gold-standard FSC=0.143) of 4.7 Å.

Modeling of the ACP-enoyl reductase domain interaction and the FAS-carboxylase metabolon

Models of *C. thermophilum* acyl carrier protein (ACP) and enoyl reductase (ER) domains were generated using Modeller 9v2 and chosen structural homologs were selected from the yeast homolog with resolved densities for both (Leibundgut et al, 2007). Additional density of ACP was observed close to the ER domain of fatty acid synthase (FAS); thus, coarse placement of the ACP was performed using CHIMERA (Pettersen et al, 2004) and subsequently fitted to the density. Manual inspection of the fit agreed with the ACP orientation with the lipid-binding domain facing the ER domain. Energy calculations using the refinement webserver were performed, the domain interaction serving as input. Energy calculations were performed as previously described (Kastritis & Bonvin, 2010; Kastritis et al, 2014). Correlation of van der Waals energy with experimentally measured equilibrium dissociation constants for known complexes was derived from (Kastritis et al, 2014).

Supplementary References

Aloy P, Russell RB (2002) Interrogating protein interaction networks through structural biology. *Proceedings of the National Academy of Sciences of the United States of America* **99:** 5896-5901

Beck M, Schmidt A, Malmstroem J, Claassen M, Ori A, Szymborska A, Herzog F, Rinner O, Ellenberg J, Aebersold R (2011) The quantitative proteome of a human cell line. *Molecular systems biology* **7:** 549

Benschop JJ, Brabers N, van Leenen D, Bakker LV, van Deutekom HW, van Berkum NL, Apweiler E, Lijnzaad P, Holstege FC, Kemmeren P (2010) A consensus of core protein complex compositions for Saccharomyces cerevisiae. *Molecular cell* **38:** 916-928

Betts MJ, Lu Q, Jiang Y, Drusko A, Wichmann O, Utz M, Valtierra-Gutierrez IA, Schlesner M, Jaeger N, Jones DT, Pfister S, Lichter P, Eils R, Siebert R, Bork P, Apic G, Gavin AC, Russell RB (2015) Mechismo: predicting the mechanistic impact of mutations and modifications on molecular interactions. *Nucleic acids research* **43:** e10

Boersema PJ, Raijmakers R, Lemeer S, Mohammed S, Heck AJ (2009) Multiplex peptide stable isotope dimethyl labeling for quantitative proteomics. *Nature protocols* **4:** 484-494

Brohee S, van Helden J (2006) Evaluation of clustering algorithms for protein-protein interaction networks. *BMC bioinformatics* **7:** 488

Brunger AT (2007) Version 1.2 of the Crystallography and NMR system. *Nature protocols* **2:** 2728-2733

Cox J, Mann M (2008) MaxQuant enables high peptide identification rates, individualized p.p.b.-range mass accuracies and proteome-wide protein quantification. *Nature biotechnology* **26:** 1367-1372

de Vries SJ, van Dijk M, Bonvin AM (2010) The HADDOCK web server for data-driven biomolecular docking. *Nature protocols* **5:** 883-897

Dominguez C, Boelens R, Bonvin AM (2003) HADDOCK: a protein-protein docking approach based on biochemical or biophysical information. *Journal of the American Chemical Society* **125:** 1731-1737

Erdős P, Rényi A (1959) On Random Graphs. I. . *Publicationes Mathematicae* **6:** 290-297

Fernandez-Recio J, Totrov M, Abagyan R (2004) Identification of protein-protein interaction sites from docking energy landscapes. *Journal of molecular biology* **335:** 843-865

Fischer L, Rappsilber J (2017) Quirks of Error Estimation in Cross-Linking/Mass Spectrometry. *Analytical chemistry* **89:** 3829-3833

Franceschini A, Szklarczyk D, Frankild S, Kuhn M, Simonovic M, Roth A, Lin J, Minguez P, Bork P, von Mering C, Jensen LJ (2013) STRING v9.1: protein-protein interaction networks, with increased coverage and integration. *Nucleic acids research* **41:** D808-815

Giese SH, Fischer L, Rappsilber J (2016) A Study into the Collision-induced Dissociation (CID) Behavior of Cross-Linked Peptides. *Molecular & cellular proteomics : MCP* **15:** 1094-1104

Gipson P, Mills DJ, Wouts R, Grininger M, Vonck J, Kuhlbrandt W (2010) Direct structural insight into the substrate-shuttling mechanism of yeast fatty acid synthase by electron cryomicroscopy. *Proceedings of the National Academy of Sciences of the United States of America* **107:** 9164-9169

Havugimana PC, Hart GT, Nepusz T, Yang H, Turinsky AL, Li Z, Wang PI, Boutz DR, Fong V, Phanse S, Babu M, Craig SA, Hu P, Wan C, Vlasblom J, Dar VU, Bezginov A, Clark GW, Wu GC, Wodak SJ et al (2012) A census of human soluble protein complexes. *Cell* **150:** 1068-1081

Jensen LJ, Julien P, Kuhn M, von Mering C, Muller J, Doerks T, Bork P (2008) eggNOG: automated construction and annotation of orthologous groups of genes. *Nucleic acids research* **36:** D250-254

Jorgensen WL, Tirado-Rives J (1988) The OPLS [optimized potentials for liquid simulations] potential functions for proteins, energy minimizations for crystals of cyclic peptides and crambin. *Journal of the American Chemical Society* **110:** 1657-1666

Kahraman A, Malmstrom L, Aebersold R (2011) Xwalk: computing and visualizing distances in cross-linking experiments. *Bioinformatics* **27:** 2163-2164

Kastritis PL, Bonvin AM (2010) Are scoring functions in protein-protein docking ready to predict interactomes? Clues from a novel binding affinity benchmark. *Journal of proteome research* **9:** 2216-2225

Kastritis PL, Rodrigues JP, Folkers GE, Boelens R, Bonvin AM (2014) Proteins feel more than they see: fine-tuning of binding affinity by properties of the non-interacting surface. *Journal of molecular biology* **426:** 2632-2652

Kirkwood KJ, Ahmad Y, Larance M, Lamond AI (2013) Characterization of native protein complexes and protein isoform variation using size-fractionation-based quantitative proteomics. *Molecular & cellular proteomics : MCP* **12:** 3851-3873

Leibundgut M, Jenni S, Frick C, Ban N (2007) Structural basis for substrate delivery by acyl carrier protein in the yeast fatty acid synthase. *Science* **316:** 288-290

Leitner A, Walzthoeni T, Aebersold R (2014) Lysine-specific chemical cross-linking of protein complexes and identification of cross-linking sites using LC-MS/MS and the xQuest/xProphet software pipeline. *Nature protocols* **9:** 120-137

Li X, Mooney P, Zheng S, Booth CR, Braunfeld MB, Gubbens S, Agard DA, Cheng Y (2013) Electron counting and beam-induced motion correction enable near-atomic-resolution single-particle cryo-EM. *Nature methods* **10:** 584-590

Liaw A, Wiener M (2002) Classification and Regression by randomForest. *R News* **2:** 18-22

Nagaraj N, Wisniewski JR, Geiger T, Cox J, Kircher M, Kelso J, Paabo S, Mann M (2011) Deep proteome and transcriptome mapping of a human cancer cell line. *Molecular systems biology* **7:** 548

Nepusz T, Yu H, Paccanaro A (2012) Detecting overlapping protein complexes in protein-protein interaction networks. *Nature methods* **9:** 471-472

Pettersen EF, Goddard TD, Huang CC, Couch GS, Greenblatt DM, Meng EC, Ferrin TE (2004) UCSF Chimera--a visualization system for exploratory research and analysis. *Journal of computational chemistry* **25:** 1605-1612

Rohou A, Grigorieff N (2015) CTFFIND4: Fast and accurate defocus estimation from electron micrographs. *Journal of structural biology* **192:** 216-221

Roy A, Kucukural A, Zhang Y (2010) I-TASSER: a unified platform for automated protein structure and function prediction. *Nature protocols* **5:** 725-738

Scheres SH (2012a) A Bayesian view on cryo-EM structure determination. *Journal of molecular biology* **415:** 406-418

Scheres SH (2012b) RELION: implementation of a Bayesian approach to cryo-EM structure determination. *Journal of structural biology* **180:** 519-530

Shannon P, Markiel A, Ozier O, Baliga NS, Wang JT, Ramage D, Amin N, Schwikowski B, Ideker T (2003) Cytoscape: a software environment for integrated models of biomolecular interaction networks. *Genome research* **13:** 2498-2504

van Zundert GC, Rodrigues JP, Trellet M, Schmitz C, Kastritis PL, Karaca E, Melquiond AS, van Dijk M, de Vries SJ, Bonvin AM (2015) The HADDOCK2.2 Web Server: User-Friendly Integrative Modeling of Biomolecular Complexes. *Journal of molecular biology*

Vizcaino JA, Deutsch EW, Wang R, Csordas A, Reisinger F, Rios D, Dianes JA, Sun Z, Farrah T, Bandeira N, Binz PA, Xenarios I, Eisenacher M, Mayer G, Gatto L, Campos A, Chalkley RJ, Kraus HJ, Albar JP, Martinez-Bartolome S et al (2014) ProteomeXchange provides globally coordinated proteomics data submission and dissemination. *Nature biotechnology* **32:** 223-226

Wasserman S, Faust K (1994) *ocial Network Analysis: Methods and Applications*: Cambridge University Press.

Williams GJ (2011) *Data Mining with Rattle and R: The Art of Excavating Data for Knowledge Discovery*: Springer.

Yang J, Yan R, Roy A, Xu D, Poisson J, Zhang Y (2015) The I-TASSER Suite: protein structure and function prediction. *Nature methods* **12:** 7-8

Appendix Fig. S1.

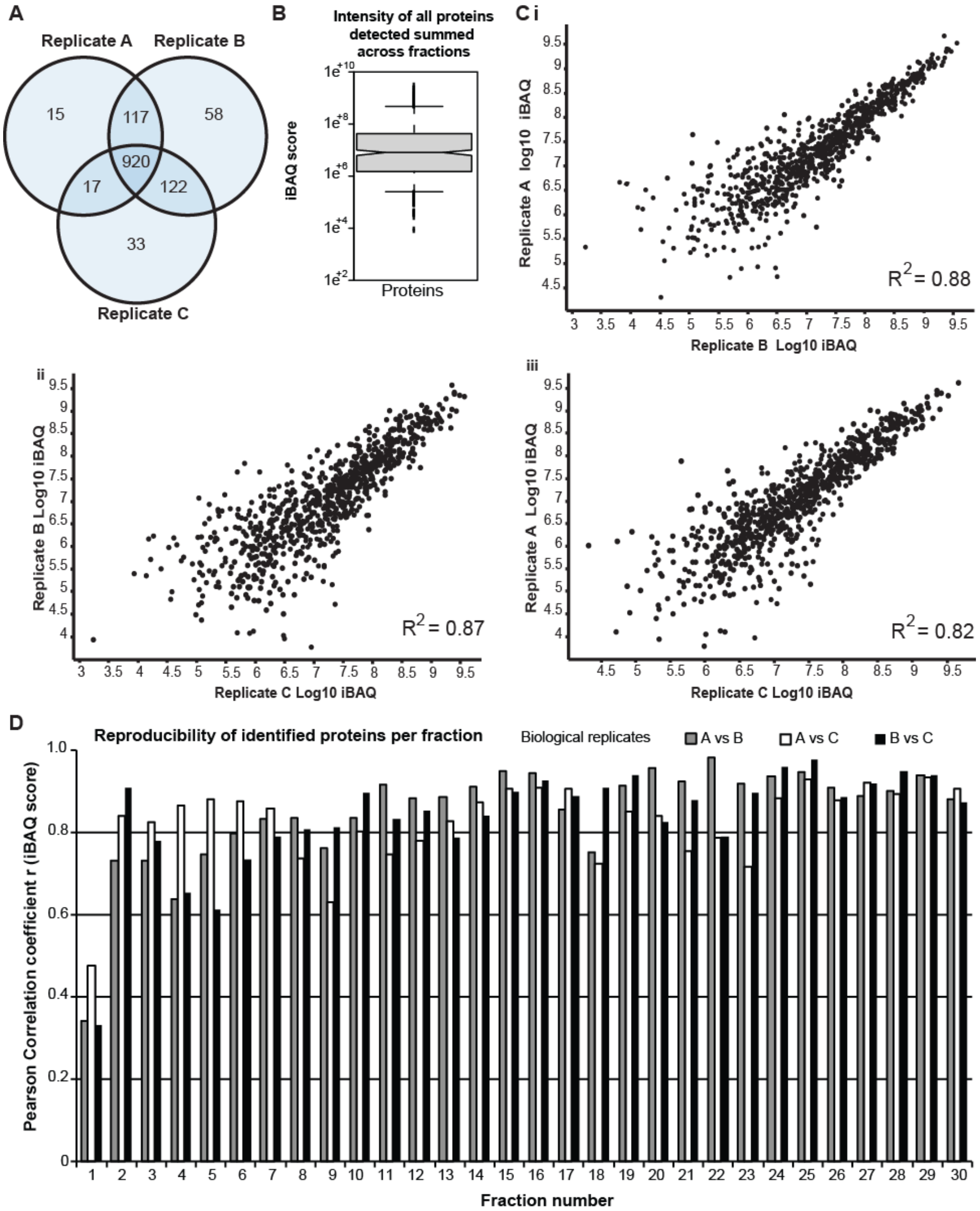

Reproducibility of quantitative LC-MS/MS analysis within biochemical fractions. **(A)** Proteins identified and their overlap between three biological replicates. **(B)** Total intensity of proteins detected (iBAQ) spans five orders of magnitude. **(C)** Reproducibility plots showing the comparison of total intensity of each protein detected (iBAQ) in the three biological replicates; A, B and C. **(D)** Reproducibility plots showing the comparison of intensity of proteins detected (iBAQ) comparing individual SEC fractions in each biological replicate.

Appendix Fig. S2.

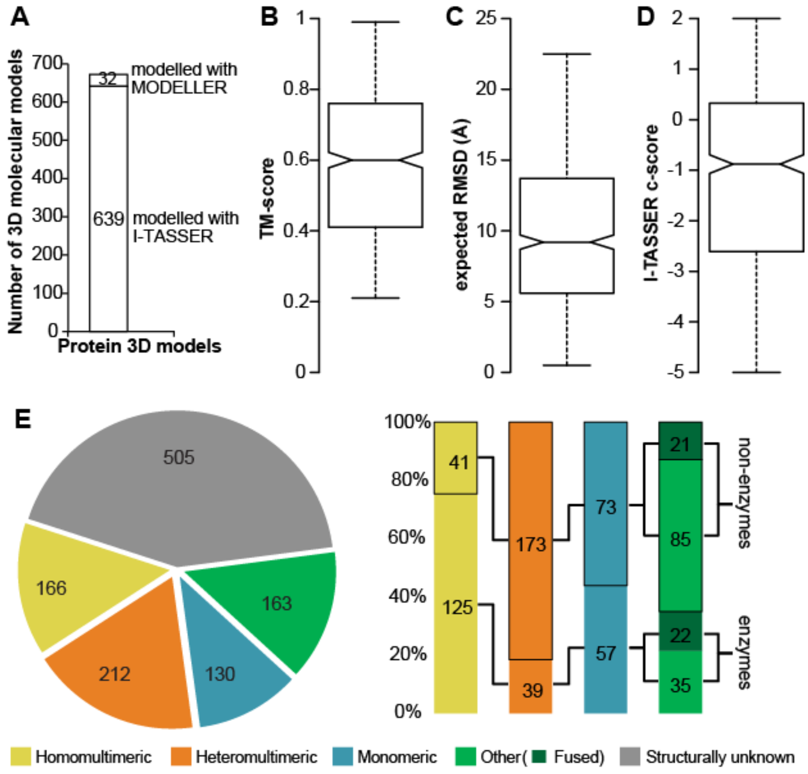

Distributions of quality measures for calculated 3D protein models. **(A)** Number of proteins sharing structural homologs that were modelled (671 in total; modeling to derive their complexes was performed by structural alignment of each model on top of its complex homolog, see **Dataset EV2**). Modeller was used when proteins failed to be modeled using I-TASSER; **(B-D)** Distribution of **(B)** expected TM-score, (C) RMSD and **(D)** C-score for all molecular models generated with I-TASSER, having >30% sequence identity, >60% sequence coverage with a homolog in the Protein Data Bank (PDB). **(E)** Statistics of identified proteins and their assignment of multimeric states in the PDB. More than half of the identified proteins acquire different stoichiometric states in the crude extract whereas those that form homomultimers mainly consist of metabolic enzymes.

Appendix Fig. S3.

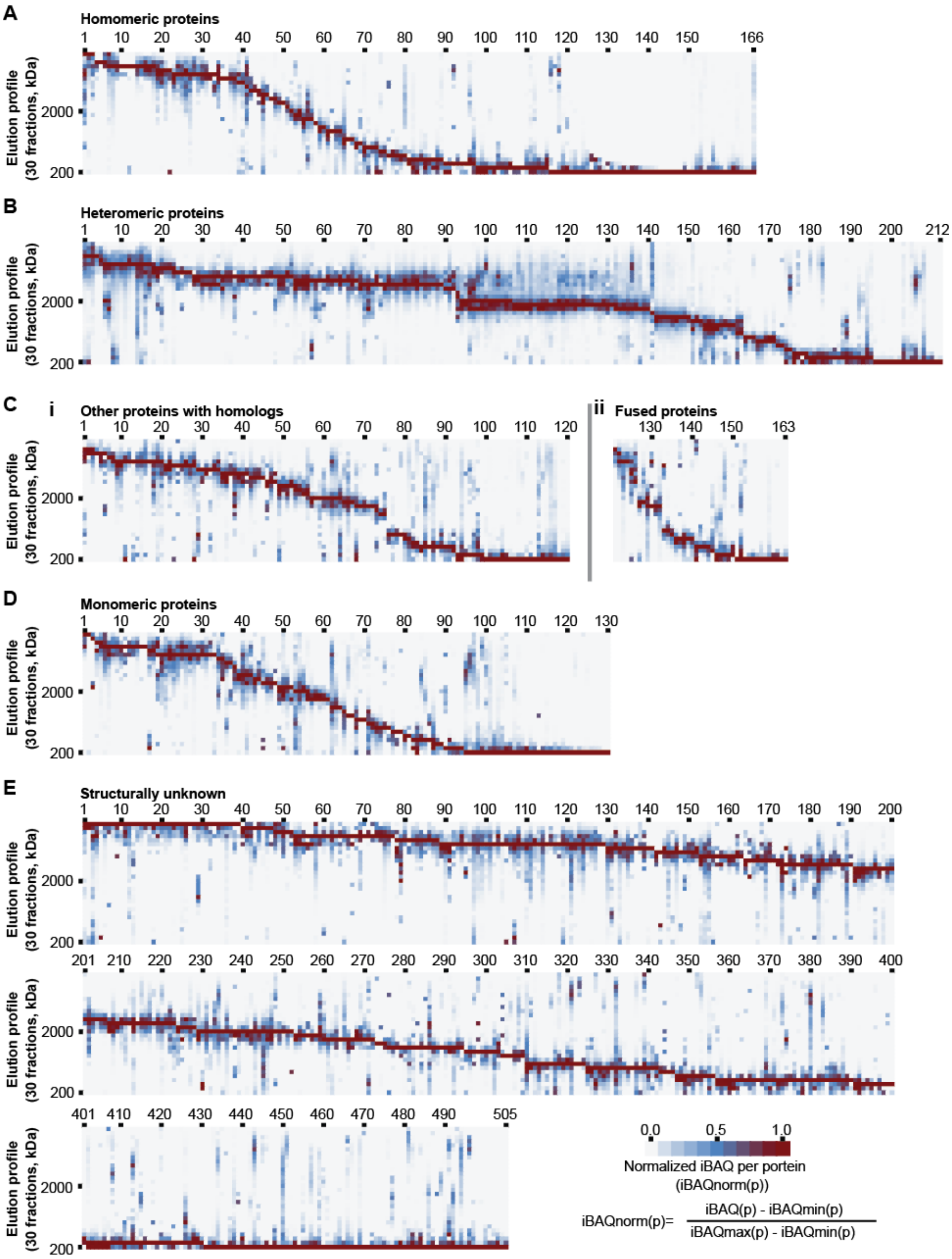

Elution profiles of all proteins categorized according to structural annotation. Elution profiles are shown as heat maps of normalized abundances (of all 1,176 proteins identified in >1 biological replicates; ranked according to the experimentally observed molecular weight). Protein IDs matching the ranks shown here are listed in **Dataset EV2**. Proteins are further categorized from A-E according to the identified PDB homologs' stoichiometry (**A**, homomultimeric complexes; **B**, heteromultimeric complexes; **C**, other proteins and fused proteins; **D**, monomeric; **E**, structurally uncharacterized).

Appendix Fig. S4.

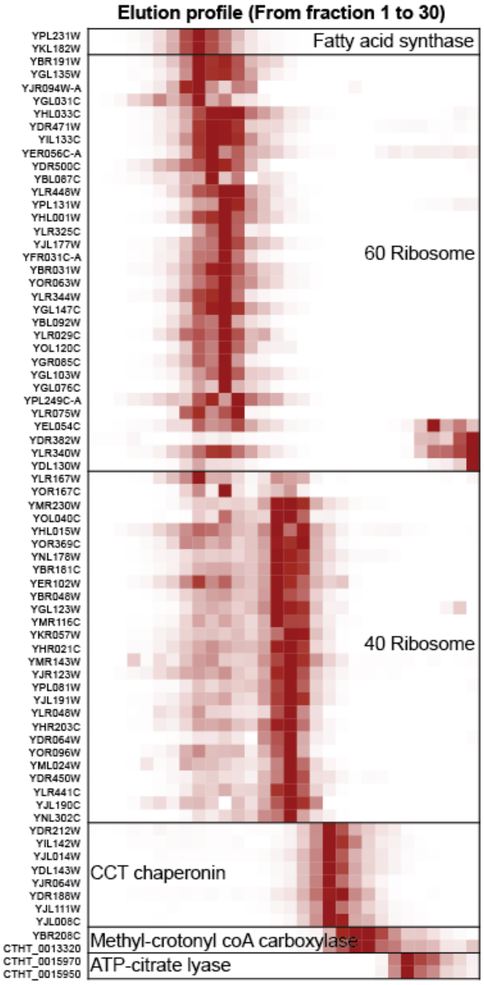

Elution profiles of protein complex subunits shown averaged in **Fig. 2A**. A clear co-elution of subunits is evident.

Appendix Fig. S5.

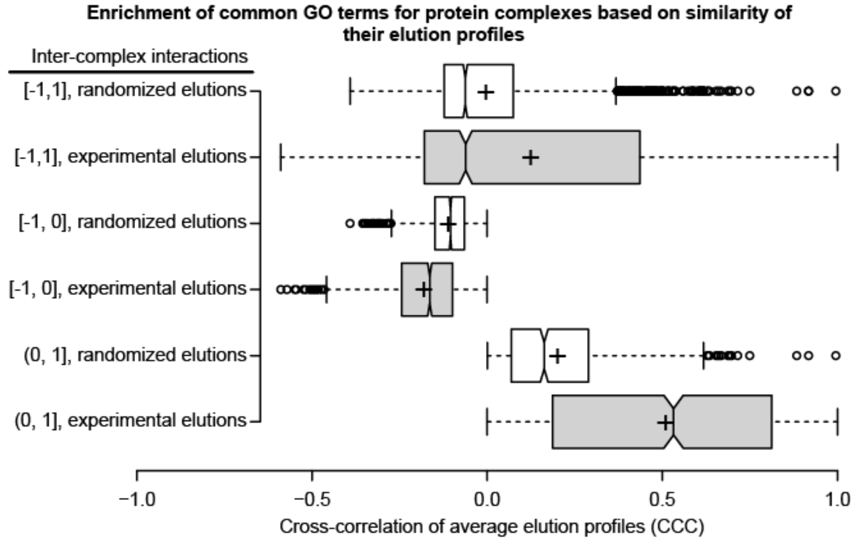

Correlation of elution profiles for protein complexes according to their GO terms. Protein elution profiles were averaged for all complexes of the protein complex benchmark (**Dataset EV2**). For each complex, GO terms were mapped and the averaged elution profiles were generated by considering the elution of all their proteins. Then, cross-correlation of their profiles were performed by grouping complexes according to their common GO terms (for the algorithm training and validation, see **Appendix Fig. S6**). Randomized elutions correspond to scrambled elution profiles for the same complexes. The higher the correlation, the more similar the elution profiles of the complexes with common GO terms. Center lines show the medians; box limits indicate the 25th and 75th percentiles as determined by R software; whiskers extend 1.5 times the interquartile range from the 25th and 75th percentiles, outliers are shown as dots; crosses represent sample means. From top boxplot to bottom, n = 1265, 987, 1601, 1879, 2866, 2866 sample points.

Appendix Fig. S6.

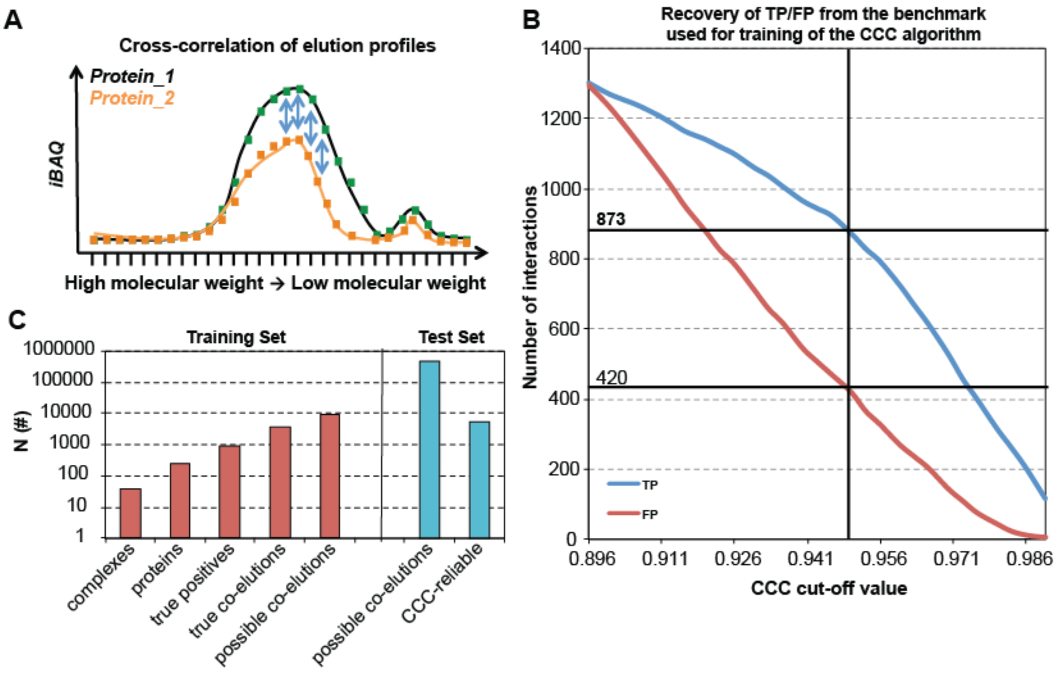

Correlation of elution profiles**. (A)** Cross-correlation function for elution profiles. A spearman correlation coefficient is calculated for each co-eluting protein pair. **(B)** FDRs at different thresholds of cross-correlation co-elution (CCC) score for the training set. **(C)** Statistics of complexes used for calibrating the CCC score for discovering protein-protein interactions and expected number of total interactions.

Appendix Fig. S7.

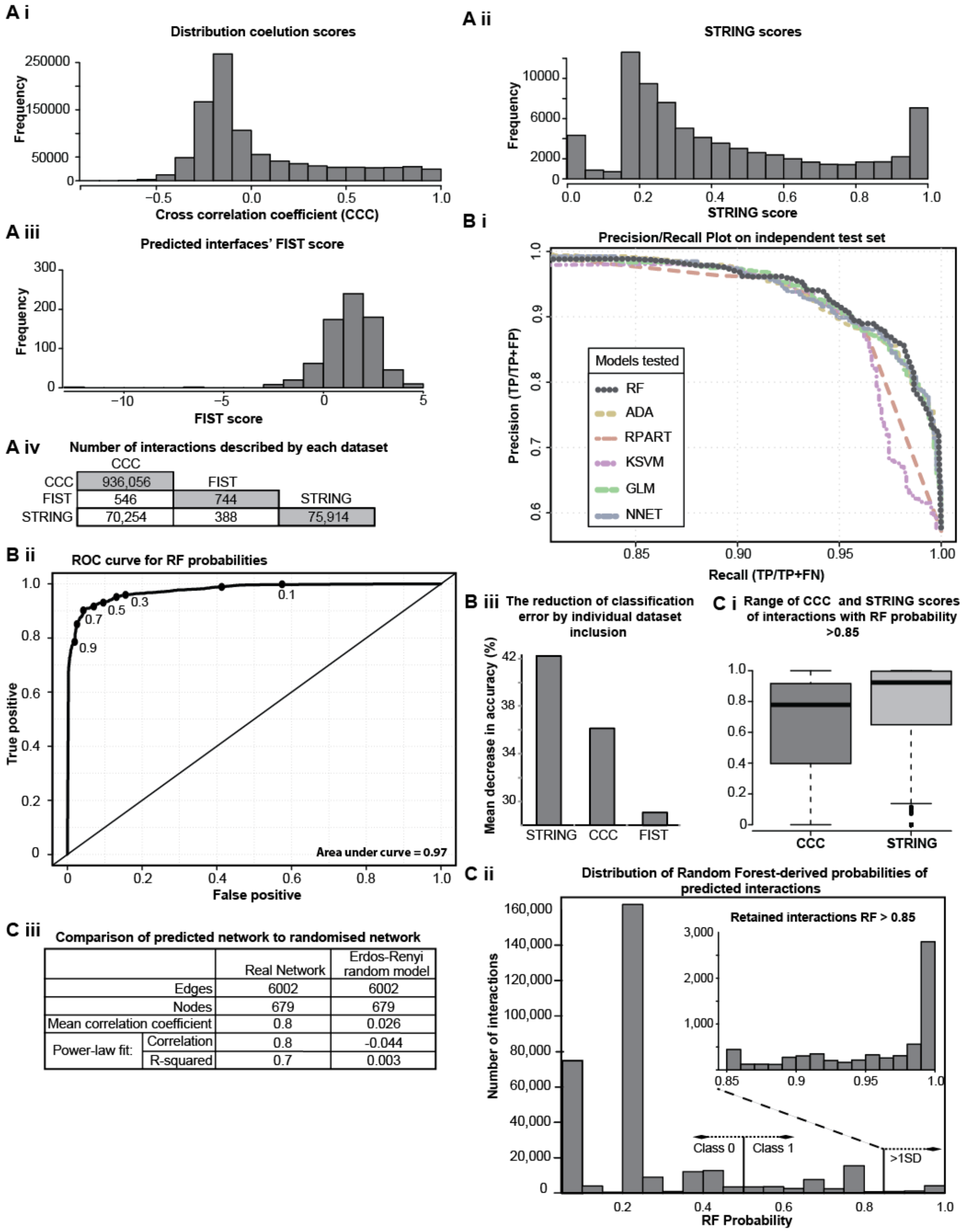

Quality measures for the reconstructed network and the communities derived using clustering **(A)** Distribution of scores **(i)** co-elution cross-correlation coefficient, **(ii)** STRING combined score, **(iii)** Mechismo Z-score from interface prediction, all of which were used to create the interaction network. **(B)** (i) Choice of machine learning approach to reconstruct the network shown in **Appendix Fig. S6B**. Precision/Recall curve showing the superior performance of Random Forests (RF) against five other tested machine-

learning models (Adaptive Boosting (ADA), Recursive Partitioning and Regression Trees (RPART), Kernel-based Support Vector Machines (KSVM), Generalized Linear Model (GLM), Neural Networks (NNET)). **(ii)** ROC curve for the RF model plotted for evaluation of the model on the independent (never seen) test dataset. The ROC curve clearly indicates prediction capacity for protein-protein interactions by the RF approach **(iii)** Evaluation of the variable importance on the model using Mean Decrease in Gini (not shown), and Mean Decrease in Accuracy (shown), which depicts how much inclusion of a predictor variable (STRING, co-elution cross-correlation coefficient (CCC), or Mechismo score) in the model reduces the prediction error. Although the most significant contributor is the external data, if CCC values from co-elution are removed, a deterioration in prediction capacity of the RF network model is observed. The smallest contribution comes from Mechismo interface score due to their low number (see **Dataset EV3**) (**C**) **(i)** Distribution of the CCC (rescaled 0-1) and the STRING combined scores covered by the RF probability threshold of 0.85-1.00. **(ii)** Distribution of RF-derived probabilities of predicted interactions, class 0 (<0.5 probability), class 1 (interaction >=0.5) with 1SD threshold of 0.85 probability for network reconstruction. The final accuracy of the dataset is within 15% of FDR; those are shown in the network in **Appendix Figs 8-10**. **(iii)** The quality of the RF-derived networks was evaluated using a randomized Erdős–Rényi model (same number of nodes and edges). Calculated network centralities showed that the network has non-random, scale-free, real-world network properties with degree distribution following the power law with correlation of 0.8 and R-squared of 0.7, compared to the randomized network correlation of -0,044, and R-squared = 0.003, and the global clustering coefficient of 0.5 vs. random network 0.026.

Appendix Fig. S8.

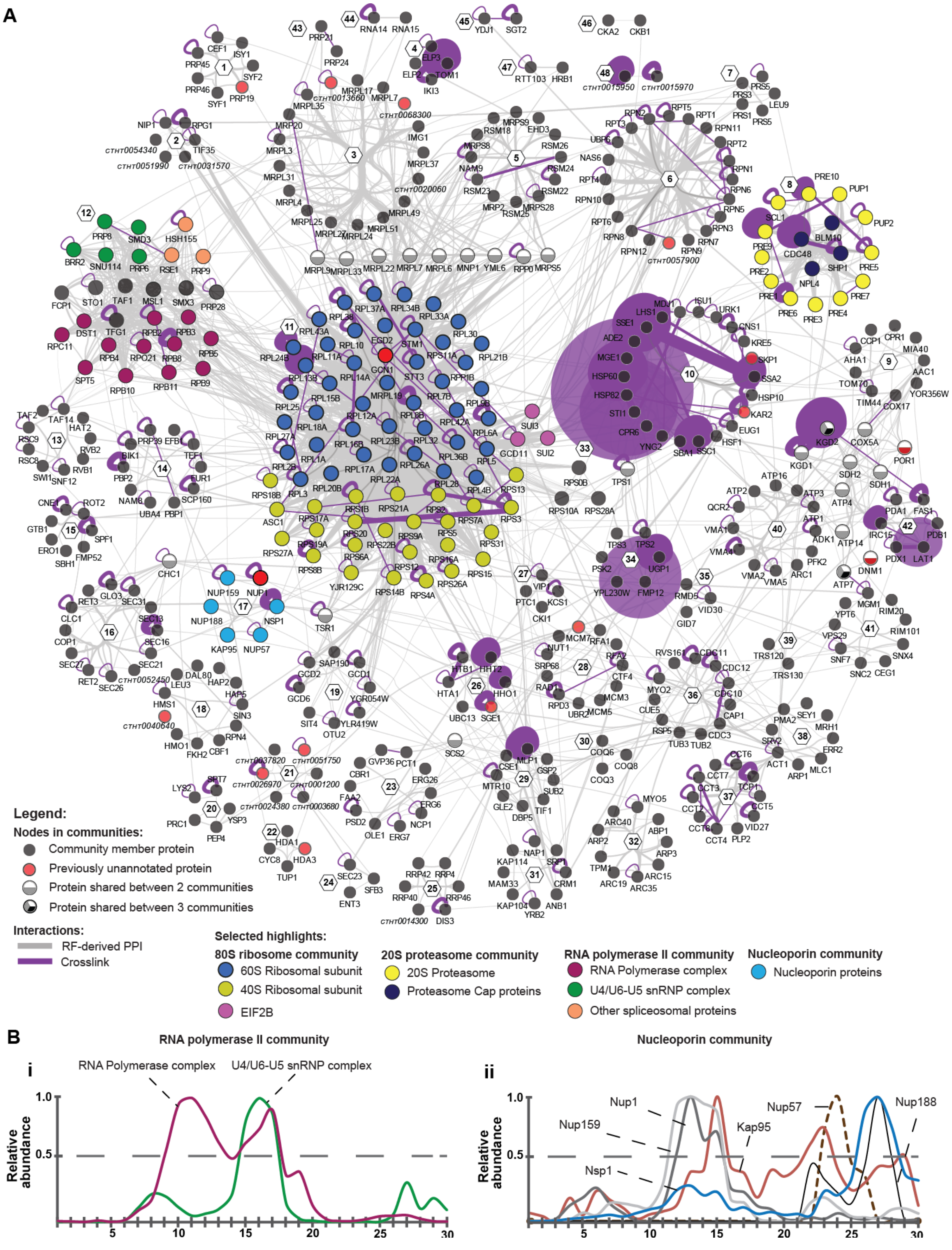

Network derived from lysate fractionation predicts protein communities. **(A)** Integration of experimental elution data, indirect interactions from STRING and predicted interaction interfaces from homologous proteins allow the creation of a high-quality network with interconnected protein complexes; mapping is shown in **Appendix Fig. S9**. Protein complex members identified ambiguously by sequence analysis but confidently taken together with the co-elution analysis are shown in red (see **Dataset EV3** for details) **(B)** Co-elution between *(i)* RNA polymerase and spiceosomal complexes is shown and also *(ii)* very transient interactions between nucleoporins are recapitulated.

Appendix Fig. S9.

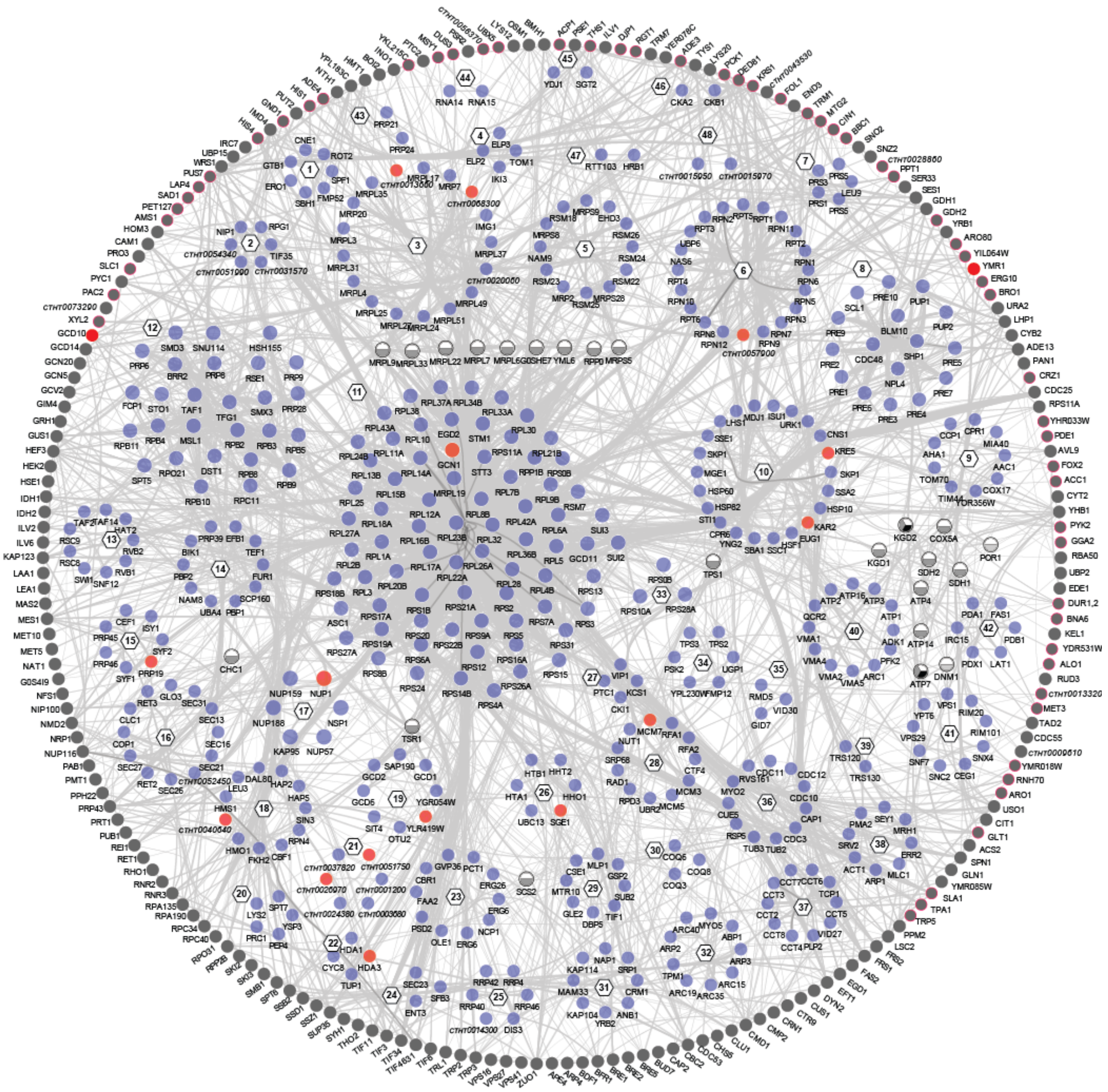

Full network figure with all proteins annotated (including not clustered into protein communities). Communities overlap with numbering from main **Fig. 3** and are as follows: 1 - Community of the NineTeen Complex; 2 - Eukaryotic initiation factor 3; 3 - 54S mitochondrial ribosome; 4 - Elongator Complex; 5 - 37S mitochondrial ribosome; 6 - 19S proteasome; 7 - PRS3-PRS5 complex; 8 - 20S Proteasome; 9 - Mitochondrial complexes community; 10 - Heat shock proteins and chaperones; 11 - 80S ribosome; 12 - RNA polymerase II and spliceosome; 13 - Chromatin remodelling proteins; 14 - Unknown community; 15 - Endoplasmic reticulum proteins; 16 - Coatomer complexes; 17 - Nucleoporins; 18 - Chromatin associated proteins; 19 - 40S ribosome binding factors; 20 - Peptidases; 21 - Cop9 signalosome; 22 - Transcriptional complexes; 23 - Lipid biosynthesis proteins; 24 - Coatomer-related proteins; 25 - Exosome; 26 - Nucleosome; 27 - Kinases; 28 - DNA associated proteins; 29 - Nuclear membrane and

import proteins; 30 - Coenzyme Q biosynthesis proteins; 31 - Nuclear importins; 32 - ARP2/3; 33 - 40S ribosome subunits; 34 - Unknown community; 35 - GID complex; 36 - Septin and Tubulin; 37 - CCT chaperonin; 38 - Cytoskeleton proteins; 39 - TRAPP complex; 40 - ATP synthase; 41 - Intracellular transport proteins; 42 - Pyruvate dehydrogenase and Oxoglutarate dehydrogenase; 43 - Spliceosomal proteins; 44 - Cleavage factor 1; 45 - SGT2-YDJ1; 46 - Protein kinase CK2 complex; 47 - Transcriptional related proteins; 48 - ATP citrate-lyase community.

Appendix Fig. S10.

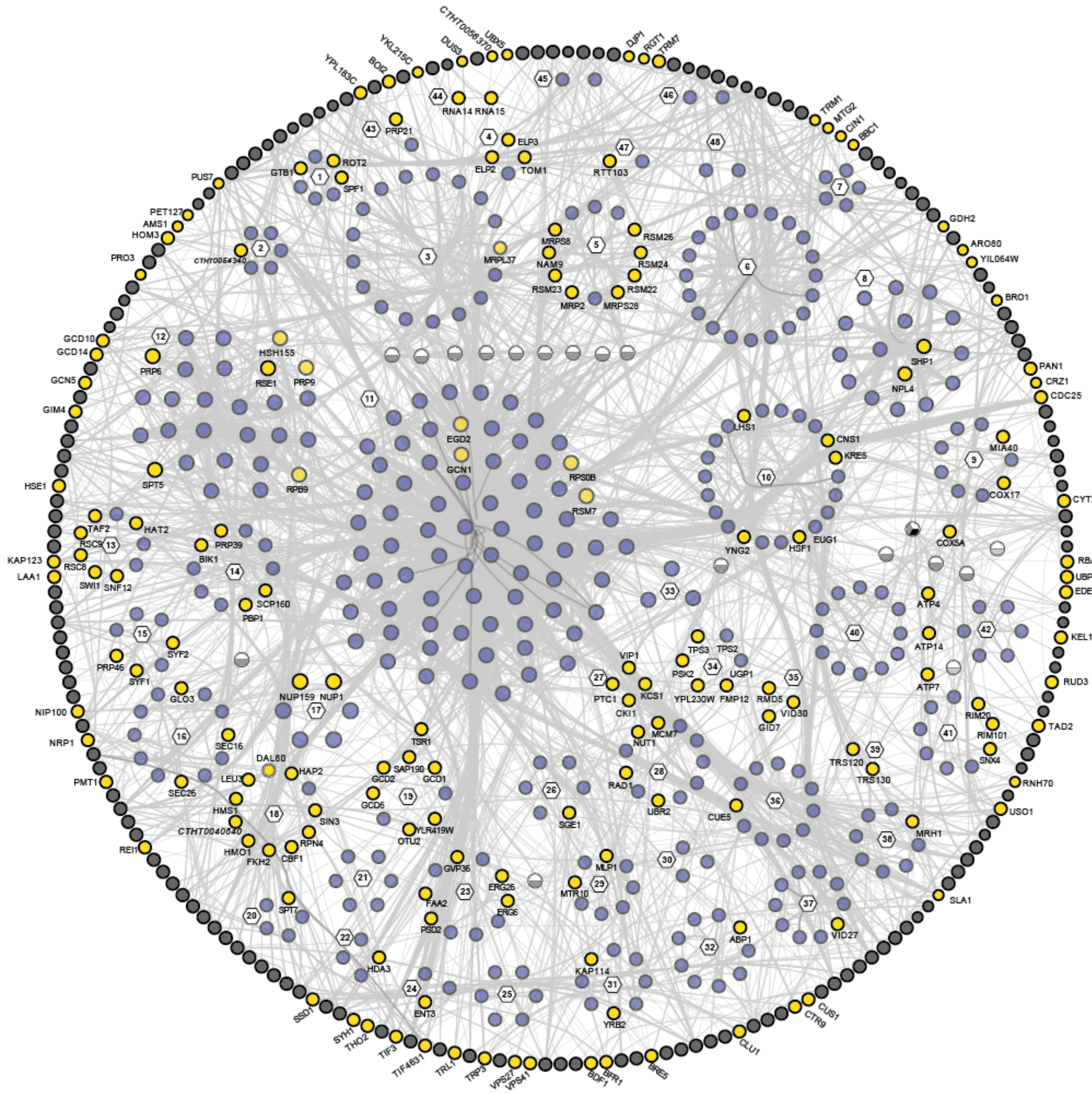

Full network with structurally uncharacterized proteins highlighted in yellow. Communities overlap with numbering described in **Figs. 3, Appendix Figs S8 and S9.**

Appendix Fig. S11.

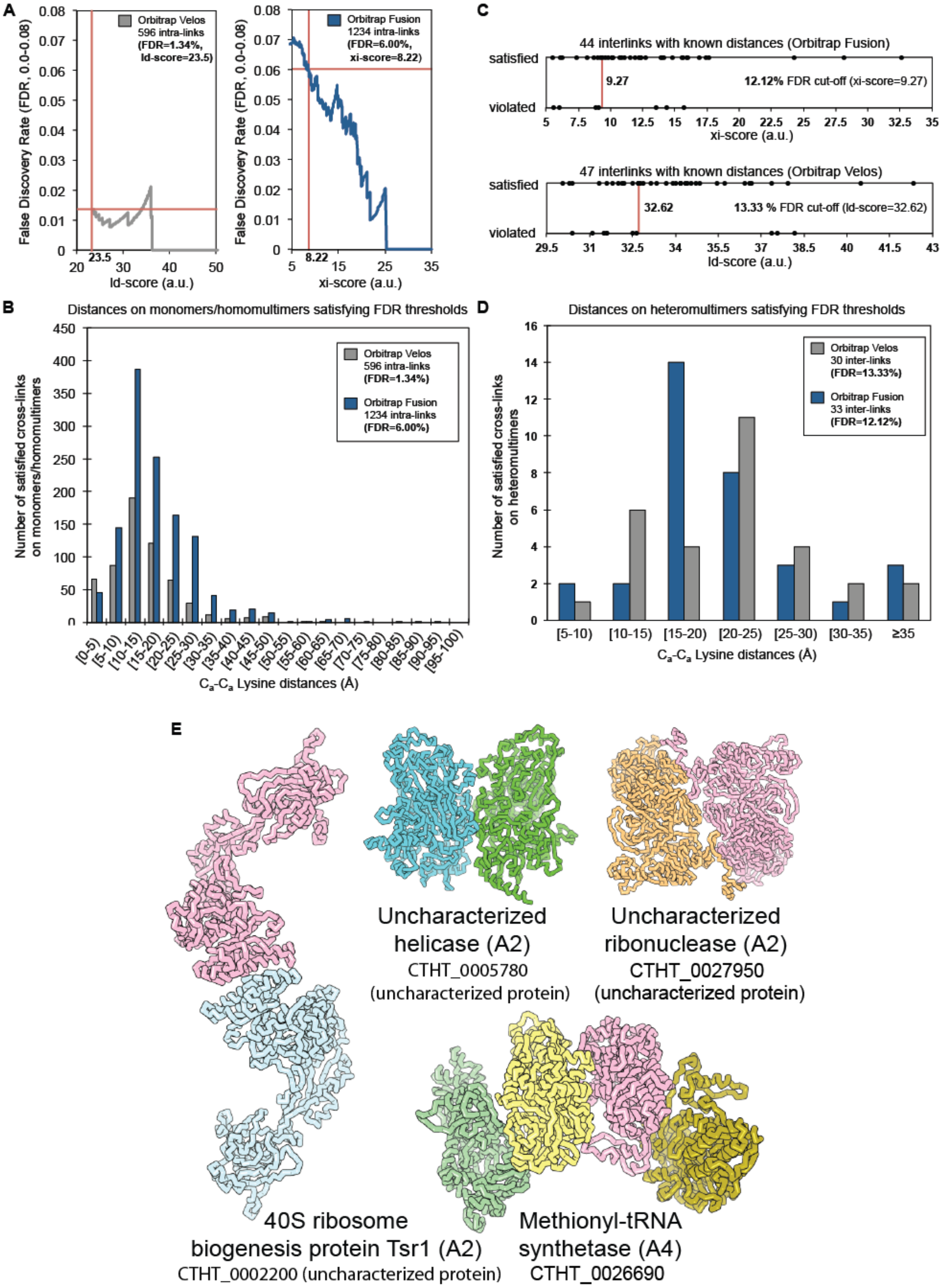

Quality calibration of intra- and inter- protein cross-links based on structural models of *C. thermophilum* proteins and complexes. **(A)** Upper panel, intra-link FDR calibration for (a) data from the two experiments using the Orbitrap Velos Pro, and (b) from the Orbitrap Lumos Fusion data. **(B)** Distance calculations of intra-protein cross-links (violated > 30 Å of distance); the log-normal experimentally determined distributions for the cross-linkers is recapitulated **(C)** Similar to (A), but corresponding to inter-protein cross-links. **(D)** Calculated distances of cross-links on protein-protein interactions; a log-normal distribution is recapitulated and few distances are violated **(E)** Docking calculations and highlighted structural models for unknown homomultimers (for details in identified novel homomultimers see **Dataset EV5** and text).

Appendix Fig. S12.

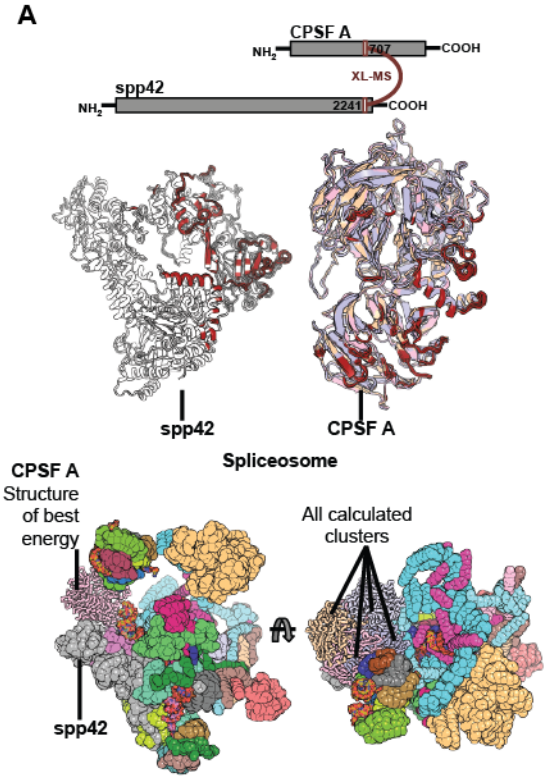

Example of docking based on inter-protein cross-links. Interface prediction based on data-driven protein docking using the cross-linking as a restraint; Below: Fitting of spp42 to the spliceosome (EMDB: 6413; PDB ID: 3JB9) after superposition of the best cluster solutions for the CPSF A-spp42 interaction.

Appendix Fig. S13.

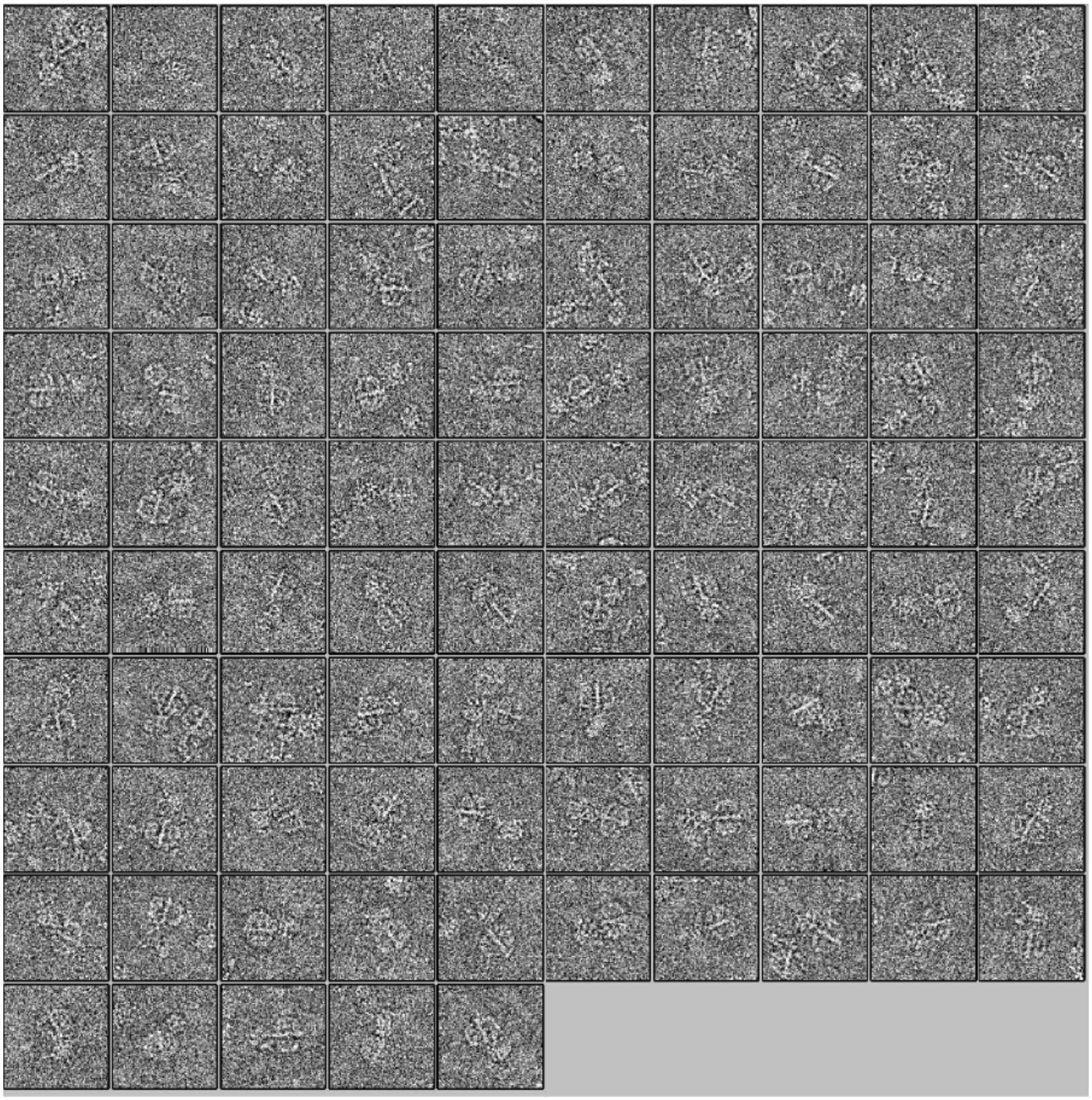

Example fatty acid synthase communities (higher-order metabolons) from cryo-EM images.

Appendix Fig. S14.

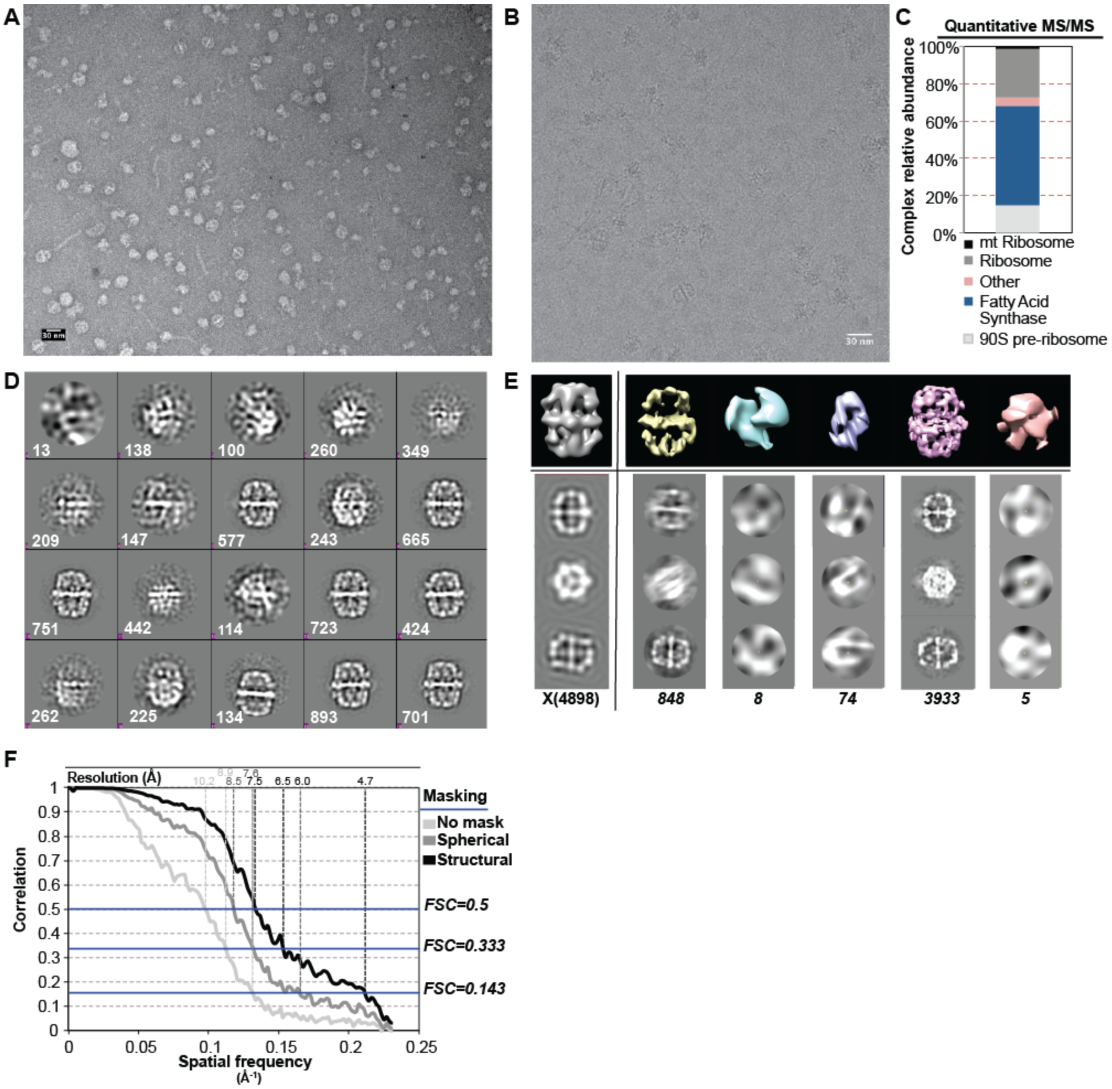

Details of ctFAS reconstruction. **(A)** Typical negative staining electron micrograph of pooled fractions 7-9 (FEI Morgagni 268(D); 100 kV, pixel size=7.1 Å; 89000 X). Fatty acid synthase (FAS) single particles are apparent. **(B)** Cryo electron micrograph of the same fraction as in (A); taken from a data set that was used to obtain the structure of ctFAS (FEI TITAN Krios; 300 kV, pixel size=2.16 Å; total dose=48 $e^{-}*Å^{2}$; 75000; defocus as determined by CTFFIND=3.4 µm). **(C)** Abundance contributions of several complexes to the same biochemical fraction as in (A, B) determined by quantitative MS; About 50% of the single particles and the total protein corresponds to FAS. **(D)** 2D class-averages of 7,370 particles of FAS and **(E)** 3D classification of 4,898 FAS particles **(F)** Fourier Shell Correlation curves with different masks; the structure obtained with a Gaussian mask created with RELION exhibits the best resolution of 4.7 Å at FSC=0.143. FSC does not reach 0 since Nyquist frequency was reached because data acquired were hard-binned 2.

Appendix Fig. S15.

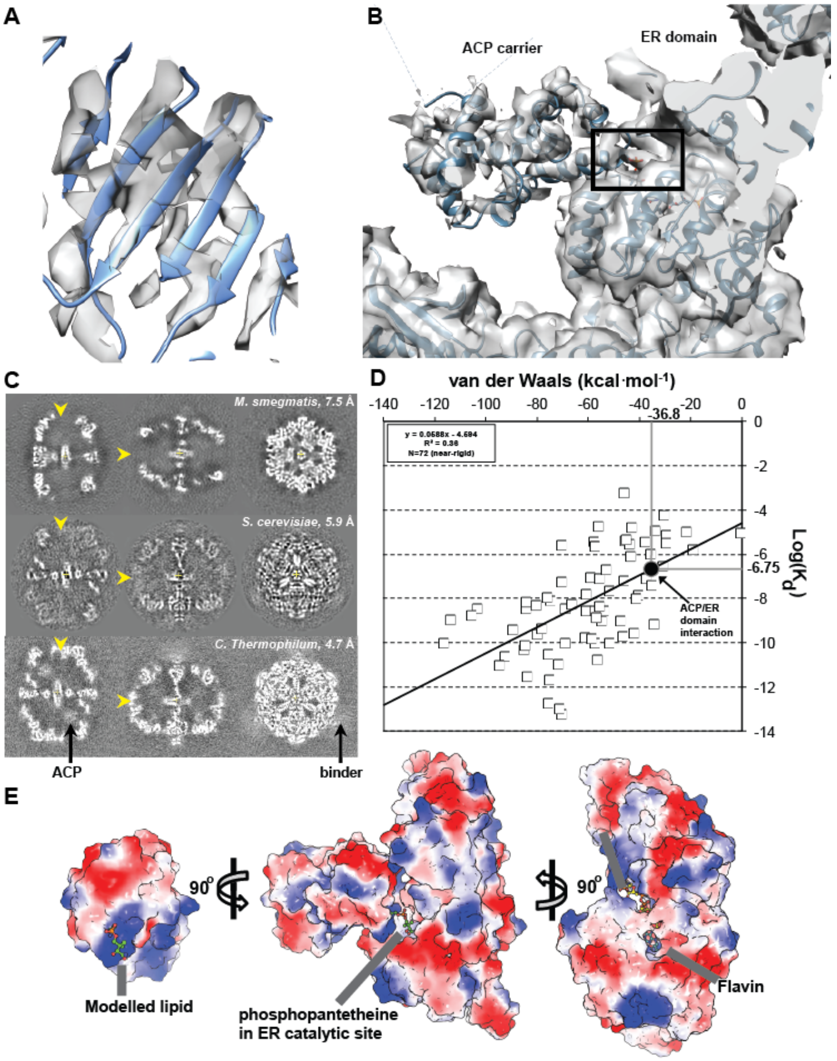

Details of ctFAS. **(A)** As expected at the given resolution of ~4,7 A, some but yet incomplete beta-strand separation is observed. **(B)** Additional density, possibly corresponding to the acyl chain within the active site between the interfaces of the acyl carrier protein (ACP) and the enoyl reductase (ER) domains is observed. (C) Comparison of cryo-EM maps of fatty acid synthase (FAS) obtained from *M. segmantis*, yeast and *C. thermophilum* (this study) that are shown as slices. ctFAS clearly exhibits additional density features in the cap region – that is the dome structure of the enzyme (region is shown with yellow arrows). The ACP domain is seen in a different position compared to the X-ray structure and additional density that cannot be explained by FAS itself is observed at the outside surface. **(D)** Prediction of the K_d for the ACP/ER interaction based on docking calculations; on the x axis, van der Waals energy calculated with HADDOCK refinement protocol is shown; on the y axis, experimentally measured binding affinities for 72 near-rigid body binders. Regression line shows predictive

capacity of the van der Waals interactions. Calculated van der Waals for the ACP/ER interaction implies the interaction to be in the μm range. **(E)** Complementation of electrostatic surface potentials for the two protein domains is apparent and comprises the major energetic determinant. Co-factors are also modeled.
